# A STEPPED WEDGE CLUSTER RANDOMIZED TRIAL OF THE STRIDE SUPERVISED WALKING PROGRAM IN VA HOSPITALS

**DOI:** 10.1093/geroni/igac059.2729

**Published:** 2022-12-20

**Authors:** Susan Hastings, Karen Stechuchak, Cynthia Coffman, Ashley Choate, Courtney Van Houtven, Virginia Wang, Cassie Meyer, Caitlin Kappler

**Affiliations:** Durham VA Health Care System, Durham, North Carolina, United States; Durham VA Health Care System, Durham, North Carolina, United States; Durham VA Health Care System, Durham, North Carolina, United States; Durham VA Health Care System, Durham, North Carolina, United States; Durham VA Health Care System, Durham, North Carolina, United States; Durham VA Health Care System, Durham, North Carolina, United States; Durham VA Health Care System, Durham, North Carolina, United States; Durham VA Health Care System, Durham, North Carolina, United States

## Abstract

In trials, hospital walking programs improved functional ability after discharge, but little evidence exists on their effectiveness under routine practice conditions or their implementation across health systems. We conducted a Type III hybrid implementation-effectiveness, stepped-wedge cluster randomized trial (SW-CRT) in 8 Veterans Affairs hospitals examining a hospital-based walking program known as STRIDE. Based on the SW-CRT design, hospitals were randomized to a sequence (timeline) for STRIDE, and additionally randomized 1:1 to receive implementation support according to the Replicating Effective Programs (REP) framework only or REP plus additional team-based communication training known as CONNECT. The study was powered to examine impact of STRIDE on discharge destination to home vs other (primary outcome); hospital length of stay (LOS) was a secondary outcome. Patient-hospitalizations in pre-STRIDE time periods (n=8167) were similar to post-STRIDE time periods (n=9070) (e.g., mean age 73, 97% male, 28–30% Black race). In adjusted models, odds of discharge to home were higher among eligible patients hospitalized in post-STRIDE time periods (OR 1.6; 95% CI 1.3–2) compared to pre-STRIDE. Findings were robust to 3 sets of sensitivity analyses. There was no difference in LOS (IRR 1.01; 95% CI 0.94,1.09). Hospitals randomized to CONNECT had higher program reach (mean 13% vs 3%) but lower daily fidelity (mean 25.7% vs 37.5%). Despite limited direct program reach, implementation of a hospital walking program was associated with higher odds of discharge to home. Implementation strategies like CONNECT that enhance teamwork and communication may improve patient access to clinical programs being implemented in new settings.

